# Cerebrospinal fluid concentrations of fluoroquinolones and carbapenems in tuberculosis meningitis

**DOI:** 10.3389/fphar.2022.1048653

**Published:** 2022-12-12

**Authors:** Nicole F. Maranchick, Mohammad H. Alshaer, Alison G. C. Smith, Teona Avaliani, Mariam Gujabidze, Tinatin Bakuradze, Shorena Sabanadze, Zaza Avaliani, Maia Kipiani, Charles A. Peloquin, Russell R. Kempker

**Affiliations:** ^1^ Infectious Disease Pharmacokinetics Lab, Emerging Pathogens Institute, University of Florida, Gainesville, FL, United States; ^2^ Department of Pharmacotherapy and Translational Research, College of Pharmacy, University of Florida, Gainesville, FL, United States; ^3^ Department of Medicine, Division of Internal Medicine, Duke University, Durham, NC, United States; ^4^ National Center for Tuberculosis and Lung Diseases, Tbilisi, Georgia; ^5^ David Tvildiani Medical University, Tbilisi, Georgia; ^6^ Department of Medicine, Division of Infectious Diseases, Emory University, Atlanta, GA, United States

**Keywords:** tuberculosis meningitis, fluoroquinolones, carbapenems, pharmacokinetics, cerebrospinal fluid, antitubercular agents

## Abstract

**Background:** Tuberculosis meningitis (TBM) is the most lethal form of TB. It is difficult to treat in part due to poor or uncertain drug penetration into the central nervous system (CNS). To help fill this knowledge gap, we evaluated the cerebrospinal fluid (CSF) concentrations of fluoroquinolones and carbapenems in patients being treated for TBM.

**Methods:** Serial serum and CSF samples were collected from hospitalized patients being treated for TBM. CSF was collected from routine lumbar punctures between alternating timepoints of 2 and 6 h after drug administration to capture early and late CSF penetration. Rich serum sampling was collected after drug administration on day 28 for non-compartmental analysis.

**Results:** Among 22 patients treated for TBM (8 with confirmed disease), there was high use of fluoroquinolones (levofloxacin, 21; moxifloxacin, 10; ofloxacin, 6) and carbapenems (imipenem, 11; meropenem, 6). Median CSF total concentrations of levofloxacin at 2 and 6 h were 1.34 mg/L and 3.36 mg/L with adjusted CSF/serum ratios of 0.41 and 0.63, respectively. For moxifloxacin, the median CSF total concentrations at 2 and 6 h were 0.78 mg/L and 1.02 mg/L with adjusted CSF/serum ratios of 0.44 and 0.62. Serum and CSF concentrations of moxifloxacin were not affected by rifampin use. Among the 76 CSF samples measured for carbapenem concentrations, 79% were undetectable or below the limit of detection.

**Conclusion:** Fluoroquinolones demonstrated high CSF penetration indicating their potential usefulness for the treatment of TBM. Carbapenems had lower than expected CSF concentrations.

## Introduction

Tuberculosis (TB) is an infectious disease caused by *Mycobacterium tuberculosis* (Mtb) and remains a major cause of mortality globally. In 2020, approximately 10 million people developed active disease and there were 1.5 million deaths, representing the first annual increase in deaths since 2005 (World Health Organization, 2021). While the lungs are the major site of TB infection, extrapulmonary disease can affect virtually all other body sites. Extrapulmonary TB accounts for approximately 15%–20% of all cases and the most severe manifestation is tuberculosis meningitis (TBM) (Sharma et al., 2021). TBM can be devastating, leading to substantial neurological impairments, paralysis, seizures, and high mortality (Bartzatt, 2011). Mortality rates during treatment for TBM are 20%–69%, with higher rates seen in cohorts of patients with drug resistant TB (Christensen et al., 2011; Dheda et al., 2017).

A major challenge in the treatment of TBM is drug delivery to the site of disease. Central nervous system (CNS) penetration of first-line TB medications including rifampin and ethambutol can be suboptimal at standard dosages, seldom exceeding minimal inhibitory concentrations (MIC) (Donald, 2010). This is especially a concern following resolution of meningeal inflammation (Thwaites et al., 2013; Roos, 2000). Second-line medications including fluoroquinolones appear to have improved penetration across the blood brain barrier, making them appealing antibiotics for the treatment of TBM (Litjens et al., 2020). Fluoroquinolones, such as levofloxacin and moxifloxacin, may be implemented into both short term and long-term regimens (Ghimire et al., 2019). Among the fluoroquinolones, moxifloxacin appears to have the highest *in vitro* activity against Mtb. However, concentrations may be most susceptible to reduction if administered with rifampin, compromising treatment efficacy (Nijland et al., 2007; Ramachandran et al., 2012). While penetration of fluoroquinolones appears to be adequate in bacterial CNS infections, additional data regarding penetration specifically in the treatment of TBM is needed.

Carbapenems are extended-spectrum beta-lactams with broad antimicrobial activity, including activity against Mtb (Jaganath et al., 2016). They are utilized for both multidrug-resistant and extensively drug-resistant TB, even though published data supporting their use is limited. To improve activity against Mtb, carbapenems are often administered with clavulanic acid, which blocks class A beta-lactamases (van Rijn et al., 2019). Data regarding imipenem and meropenem penetration into the CSF suggests adequate exposure to eradicate most pathogens, especially in the presence of CNS inflammation (Zhanel et al., 2007; Craig, 1997). However, data is limited regarding the CNS penetration of carbapenems in TBM (Jaganath et al., 2016).

To further understand the exposure of fluoroquinolones and carbapenems in TBM, our objective was to describe and compare serum and CSF concentrations of fluoroquinolones (levofloxacin, moxifloxacin, and ofloxacin) and carbapenems (imipenem and meropenem) among persons treated for TBM.

## Materials and methods

### Setting and participants

This prospective study enrolled participants at the National Center for Tuberculosis and Lung Diseases (NCTLD) in Tbilisi, Georgia. The NCTLD includes an inpatient ward for patients with TBM and directly observed therapy clinics for ambulatory care. Study participants were selected from a larger prospective cohort evaluating clinical outcomes among persons treated for TBM (Smith et al., 2021). Written formal consent was obtained from all study participants. The study was approved by the institutional review boards of the NCTLD (IRB# IORG0009467), the University of Florida, Gainesville, FL, United States, and Emory University, Atlanta, GA, United States.

All patients underwent a lumbar puncture and CSF diagnostic testing, including acid-fast bacilli (AFB) staining, molecular testing with Xpert^®^ MTB/RIF assay (Cepheid, United States) and AFB culture on a Lowenstein-Jensen solid and Mycobacterial Growth Indicator Tube liquid media. To identify drug resistance to rifampin and isoniazid, a Genotype^®^ MTBDR*plus* (Hain-Lifescience, Germany) assay was performed on positive cultures. Serial lumbar punctures were performed at 7, 14, 28 days, and then monthly after treatment initiation per standard of care. With regards to treatment and dosing, WHO guidance was followed and treatment regimens were based on the Georgian National TB Program 2015 guidelines, as outlined previously (Smith et al., 2021). Both levofloxacin and moxifloxacin were given by mouth (levofloxacin dose was either 750 mg or 1000mg, moxifloxacin 400 mg or 1000 mg). Ofloxacin was administered as 800 mg intravenously every 12 h. Meropenem and imipenem were administered intravenously as 1000 mg every 12 h. Multiple participants received more than one study drug of interest from the same class, but not concurrently. All participants received multidrug regimens for TBM, although only fluoroquinolones and carbapenems were analyzed in the present study (Smith et al., 2021). For MDR-TBM, regimens were chosen based upon Georgian National TB guidelines, and patients received regimens including carbapenems, injectable agents (such as amikacin), linezolid, and novel/repurposed anti-TBM agents such as clofazimine, delamanid, and bedaquiline (Smith et al., 2021). Additionally, all participants received adjunctive dexamethasone therapy for 6–8 weeks. Participants were hospitalized for treatment until clinical improvement such that they could be treated in an outpatient setting (Smith et al., 2021; Kempker et al., 2022).

### Drug sample collection and quantification

Participants enrolled in the pharmacokinetic (PK) study had both serum and CSF samples collected at baseline and at approximately 7, 14, and 28 days following initiation of treatment. Monthly samples were subsequently collected during hospitalization for up to 112 days. Participants had two- and 6-h samples collected following drug administration from both serum and CSF. The CSF sampling was alternated between two- and 6-h concentrations to assess for early and delayed penetration. Rich serum sampling for non-compartmental analysis (NCA) was performed at 28 days, and the collection timepoints were 0, 2, 4, 8, 12, and 24 h after drug administration with slight variations for persons receiving bedaquiline (0, 2, 6, 12, 24, 48, 72 h) and delamanid (0, 1, 2, 4, 6,8 and 12 h). Following serum and CSF collection, samples were centrifuged then stored at −80°C at the NCTLD until shipping to the Infectious Disease Pharmacokinetic Laboratory (IDPL) at the University of Florida, with the cold chain remaining intact throughout. Total drug concentrations were quantified using validated liquid chromatography tandem mass spectrometry assays. Serum curve was used for the serum samples, while artificial CSF curve was used for CSF samples to match CSF matrix. The plasma and CSF detection ranges for levofloxacin, moxifloxacin, and ofloxacin were 0.2–15 mg/L and 2–100 mg/L for meropenem and imipenem. Intra- and inter-batch precision and accuracy were <10%. Samples below the limit of quantification (BLQ) were assigned a value of “0” for analysis.

### Data management

NCA was performed using Phoenix WinNonlin (Certara, v8.3) to determine maximum serum concentration (C_max_), time to maximum concentration (T_max_), elimination rate constant (K_e_), half-life (t_1/2_) and area under the concentration-time curve over 24 h (AUC_0-24_). Serum protein binding rates for levofloxacin (31%), moxifloxacin (40%), ofloxacin (32%), imipenem (20%), and meropenem (2%) were obtained from previously published literature and used to estimate free serum drug concentrations (Craig, 1997), (Lamp et al., 1992; Janssen Pharmaceuticals and Inc, 1996; Pickerill et al., 2000; Ortho-McNeil-Janssen Pharmaceuticals and Inc, 2008; Bolon, 2009; Salmon-Rousseau et al., 2020). We assumed there was no protein binding in the CSF (i.e., 100% free drug) (Bonati et al., 1982; de Lange, 2013). To assess CSF penetration, a ratio of CSF to serum concentrations was calculated for matching time points for participants. Adjusted CSF to serum ratios were calculated to account for serum protein binding. Samples BLQ or zero were excluded in the ratio calculations.

Continuous data were presented as median and interquartile range, and categorical data as count and percentage. Student t-tests were used to assess for differences in drug concentrations at 2 and 6 h, differences in the CSF to serum ratios between 2 and 6 h, and compare CSF concentrations from baseline over the duration of therapy to the final drawn sample for each drug at two and 6-h timepoints. JMP Pro v16.1 (JMP, Cary, NC, United States) was used for statistical analysis.

## Results

**FIGURE 1:**
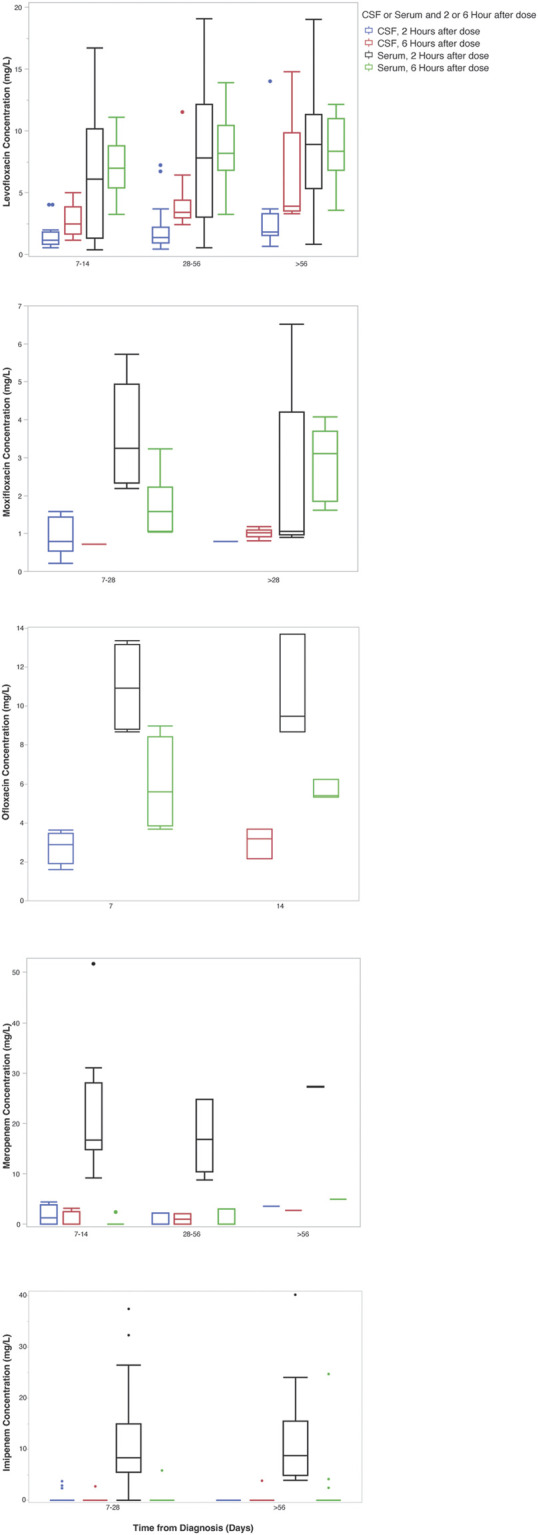
Carbapenem and fluoroquinolone CSF and serum concentrations over time from diagnosis. *The x-axis indicates the time in days from the start of any anti-tuberculosis treatment. Boxes indicate the median and interquartile range of the samples.

Twenty-two participants were enrolled in the PK study, all of whom received a fluoroquinolone and/or carbapenem. Over half (55%) of enrolled patients were female and the median (IQR) age was 38 (29.3–47.8) years. Additional information regarding baseline demographics, comorbidities, and relevant clinical measures including CSF composition are included in Tables 1, 2. Of note, CSF white blood cell count declined over the first 28 days of treatment (Table 2). Trends in fluoroquinolone and carbapenem CSF and serum concentrations after diagnosis of TBM can be visualized in (Figure 1).

**TABLE 1:** Participant demographics, clinical characteristics, and clinical outcomes (*n* = 22).

Characteristic	*n* (%), or median (IQR)
Male	10 (45%)
Age, years	38 (29.3–47.8)
BMI, kg/m^2^	24.5 (22.2–26.5)
HIV Positive^#^	1 (5%)
Chronic Hepatitis C^¥^	3 (14%)
Prior TB treatment	7 (47%)
IVDU	2 (9%)
**Clinical Characteristics**	
Baseline GCS	15 (9–15)
TB Meningitis Confirmation	8 (36%)
**TB Treatment Category**	
Susceptible	16 (72.7%)
MDR-TB defined clinically^§^	3 (13.6%)
MDR-TB confirmed with culture	3 (13.6%)
**Clinical Outcomes at End of the Study**	
Treatment Completed	21 (95%)
Treatment Failure	1 (5%)
Seizures Reported with Carbapenems	0 (0%)

HIV, human immunodeficiency virus; CSF, cerebral spinal fluid; TB, tuberculosis; IVDU, intravenous drug use; GCS, glascow coma scale; MDR-TB, multidrug resistant TB (resistance to at least both isoniazid and rifampicin).

#One patient received Atripla^®^ (efavirenz, emtricitabine, tenofovir disoproxil) during TBM treatment, with minimal impact on study medications expected.

¥No patients received hepatitis C medications simultaneously with TB treatment.

§Among patients with clinically confirmed MDR-TB, one had a known MDR-TB contact and two previously received TB treatment.

**TABLE 2:** Cerebrospinal fluid analysis*.

Timepoint (Days)	WBC (cells/μL)	Lymphocyte %	Neutrophil %	Total protein (mg/dl)
0	133 (143)	92 (9.5)	5 (4.8)	99 (66)
7	86.5 (192.5)	90 (13)	4 (5)	66 (33)
14	71 (45)	86 (32)	3 (9)	33 (33)
28	33 (23)	70 (94)	0.5 (2)	33 (27)
56	38 (39)	69.5 (94.3)	0 (11.3)	33 (33)
84	44 (28)	92.5 (20.8)	2.5 (4.5)	49.5 (57.8)
112	32 (20.5)	88 (51)	2 (3)	66 (66)

*Data represented as Median (IQR). WBC, white blood count.

### Fluoroquinolones

A total of 21 participants received levofloxacin during their treatment, resulting in 45 matching CSF and serum samples at 2 h and 38 at 6 h (Table 3). Median (IQR) total serum concentrations for levofloxacin at 2 and 6 h were 7.36 mg/L (2.83–11.21) and 7.68 mg/L (5.75–10.11), while total CSF concentrations at 2 and 6 h were 1.34 mg/L (0.91–1.98) and 3.36 mg/L (2.43–4.03). C_max_ concentrations through NCA were 11.62 mg/L (8.64–13.12), with a T_max_ of 4 h (Table 4). Median CSF to serum ratios adjusted for protein binding were higher at six *versus* 2 h (0.63 vs 0.41, *p* = 0.05). Following TBM diagnosis, levofloxacin CSF concentrations increased significantly from baseline at both 2 h (*p* = 0.02) and 6 h (*p* = 0.002) (Table 5).

**TABLE 3:** Fluoroquinolone serum and cerebrospinal fluid concentrations.

Drug	Time from administration to sample collection (hours)	Participants	CSF samples	CSF drug concentration (mg/L) (median, IQR)	Serum samples	Serum drug concentration (mg/L) (median, IQR)	Number of matching samples	Median CSF/serum concentration	Median adjusted CSF/serum concentration
Levofloxacin	2	21	53	1.34 (0.91–1.98)	87	7.36 (2.83–11.21)	45	0.28 (0.13–0.73)	0.41 (0.19–1.06)
	6	21	38	3.36 (2.43–4.03)	68	7.68 (5.75–10.11)	38	0.44 (0.33–0.54)	0.63 (0.47–0.78)
Moxifloxacin	2	10	10	0.78 (0.63–1.40)	17	2.74 (1.65–4.84)	10	0.26 (0.17–0.31)	0.44 (0.28–0.52)
	6	6	6	1.02 (0.78–1.06)	12	1.91 (1.56–3.28)	6	0.37 (0.30–0.50)	0.62 (0.49–0.84)
Ofloxacin	2	5	6	2.22 (0.93–3.11)	7	9.47 (8.67–13.36)	4	0.25 (0.19–0.30)	0.37 (0.29–0.44)
	6	4	3	3.20 (2.17–3.69)	7	5.40 (4.43–6.75)	3	0.51 (0.41–0.68)	0.75 (0.60–1.01)

*CSF, cerebrospinal fluid; IQR, interquartile range.

**TABLE 4:** Pharmacokinetic parameters for fluoroquinolones and carbapenems in participants*.

	Dose (mg)	C_max_ (mg/L)	T_max_ (hr)	K_e_ (1/Hr)	Half-life (hr)	AUC_0-24_ (Hr*mg/L)
Levofloxacin (*n* = 20)	750	11.62 (8.64–13.12)	4 (2–5.5)	0.15 (0.12–0.20)	4.79 (3.54–5.97)	94.85 (84.70–127.26)
Moxifloxacin (*n* = 5)	400	4.04 (2.78–5.34)	2 (1.5–3)	0.14 (0.08–0.16)	4.95 (4.31–8.93)	33.86 (22.21–44.57)
Ofloxacin (*n* = 7)	800	9.47 (8.67–13.36)	2 (2–2)	0.17 (0.12–0.20)	4.12 (3.52–5.71)	122.4 (101.98–155.68)
Imipenem (*n* = 11)	1000	20.8 (7.41–37.43)	1.5 (0.5–2)	0.68 (0.50–1.09)	1.02 (0.64–1.40)	65.80 (39.78–144.00)
Meropenem (*n* = 6)	1000	16.44 (10.00–25.46)	2 (2–2)	0.37 (0.21–0.73)	1.03 (0.95–3.36)	22.91 (0–136.14)

*Data represented as Median (IQR). Some participants had multiple instances of rich sampling, which are included as part of n. C_max_, maximum concentration of drug; T_max_, time to achieve maximum drug concentration in serum; K_e_, elimination rate constant; AUC_0-24_, area under the concentration time curve from 0 to 24 h.

**TABLE 5:** Analysis of increased CSF concentrations and days on treatment for fluoroquinolones.

		Days on treatment	
Drug	CSF concentration	Parameter estimate	*p*-value
Levofloxacin	2-Hour	0.02	0.02*
	6-Hour	0.04	0.002*
Moxifloxacin	2-Hour	−0.0008	0.90
	6-Hour	0.002	0.39
Ofloxacin	2-Hour	0.28	0.04*
	6-Hour	-	-

*Indicates significant value, <0.05. CSF, cerebrospinal fluid. Ofloxacin CSF concentrations at 6 h did not have enough time points to assess concentration over time.

Ten participants received moxifloxacin during treatment, 6 (60%) following a change in therapy from levofloxacin. There were 10 and six matching CSF and serum samples at 2 and 6 h, respectively. Median (IQR) total serum concentrations at 2 and 6 h were 2.74 mg/L (1.65–4.84) and 1.91 mg/L (1.56–3.28), while median total CSF concentrations at 2 and 6 h were 0.78 mg/L (0.63–1.40) and 1.02 mg/L (0.78–1.06) (Table 3). C_max_ concentrations through NCA were 4.04 mg/L (2.78–5.34), with a T_max_ of 2 h (Table 4). Median CSF to serum concentrations adjusted for protein binding increased from 2 to 6 h (0.44 vs 0.62, *p* = 0.05). Following TBM diagnosis and treatment initiation, moxifloxacin in CSF trended towards increased concentrations, but this was not statistically significant Among the participants receiving moxifloxacin, seven received concomitant rifampin, yet no difference in moxifloxacin serum AUC_0-24_ were seen between patients receiving rifampin *versus* those not receiving rifampin (32.86 vs 33.38 Hr*mg/L, *p* = 1.00). Additionally, CSF concentrations were not significantly different between patients receiving rifampin *versus* not receiving rifampin at 2 h (0.78 vs. 0.67 mg/L, *p* = 0.78) or 6 h (1.02 vs 0.72 mg/L, *p* = 0.72).

Five participants received ofloxacin, with four matched CSF to serum samples at 2 h and three at 6 h. Median (IQR) total serum concentrations at 2 and 6 h were 9.47 mg/L (8.67–13.36) and 5.40 mg/L (4.43–6.75), while total CSF concentrations at 2 and 6 h were 2.22 mg/L (0.93–3.11) and 3.20 mg/L (2.17–3.69) (Table 3). Median C_max_ concentrations through NCA were 9.47 mg/L (8.67–13.36), with a T_max_ 2 h (Table 4). Median CSF to serum concentrations adjusted for protein binding were higher at 6 h *versus* 2 h (0.76 vs 0.37, *p* = 0.03) (Table 3). Ofloxacin CSF concentrations at 2 h increased significantly from baseline (*p* = 0.04) whereas concentrations at the 6-h time-point did not (*p* = 0.28) (Table 5).

### Carbapenems

There were 11 participants who received imipenem resulting in three matched CSF to serum samples at 2 h and two at 6 h. For meropenem, six participants with six matched CSF to serum samples at 2 h and one matched sample at 6 h were available. With the exception of 2-h serum samples, a substantial number of samples were undetectable or BLQ (<2 mg/L) for both serum and CSF (Table 6). 12/15 (80%) of meropenem total serum concentrations at 6 h were undetectable or BLQ in addition to 6/12 (50%) and 5/9 (56%) meropenem total CSF concentrations at 2 and 6 h 39/43 (91%) imipenem total serum concentrations at 6 h were undetectable or BLQ in addition to 28/32 (88%) and 21/23 (91%) total CSF concentrations at 2 and 6 h, respectively. Meropenem had more detectable levels in the CSF than imipenem.

**TABLE 6:** Carbapenem serum and cerebrospinal fluid concentrations.

Drug	Time from administration to sample collection, h	Participants	CSF samples	CSF drug concentrations BLQ or 0	CSF drug concentration (mcg/ml) (median, IQR)	Serum samples	Serum drug concentrations BLQ or 0	Serum drug concentration (mcg/ml) (median, IQR)	Number of matching samples (excluding BLQ and 0)	Median CSF/serum concentration	Median adjusted CSF/serum concentration
Imipenem	2	11	32	28	0 (0–0)	52	1	8.33 (5.36–15.09)	3	0.47 (0.36–0.48)	0.59 (0.46–0.60)
	6	11	23	21	0 (0–0)	43	39	0 (0–0)	2	1.01 (0.47–1.56)	1.27 (0.59–1.95)
Meropenem	2	6	12	6	0 (0–3.30)	20	0	17.42 (14.82–26.71)	6	0.15 (0.12–0.29)	0.15 (0.13–0.30)
	6	6	9	5	0 (2.54)	15	12	0 (0–0)	4	0 (0–0.42)	0 (0–0.43)

*CSF, cerebrospinal fluid; IQR, interquartile range; BLQ, below limit of quantification.

## Discussion

Our findings demonstrate a high CSF penetration of fluoroquinolone antibiotics and provide support for their consideration in the treatment of TBM. In particular, the CSF concentrations of levofloxacin were high and above the Mtb susceptibility cutoff in most samples tested, supporting its ability to achieve effective concentrations in TBM. Our study also provides novel data on the CSF concentrations of antibiotics over time which unexpectedly trended higher with the fluoroquinolones despite decreasing CSF inflammation, providing data that continued moderate-high CSF concentrations can be obtained during treatment. While current clinical trial data have not shown an ability of fluoroquinolones to improve outcomes among patients treated for drug-susceptible TBM, our study provides key pharmacokinetic data for their continued study in clinical trials and use in drug-resistant TBM (Heemskerk et al., 2016; Huynh et al., 2022). To the contrary, our study revealed that the carbapenems did not achieve adequate concentrations in our patient cohort, questioning their utility for patients with TBM.

All fluoroquinolones in our study had median serum C_max_ values within normal ranges (8–12 mg/L for levofloxacin and ofloxacin, 3–5 mg/L for moxifloxacin) indicating typical systemic exposure (Alsultan and Peloquin, 2014). Our finding of increasing CSF concentrations from 2 to 6 h demonstrates delayed penetration which has important implications. In the case of levofloxacin, this delay may have been in part from delayed oral absorption as median serum concentrations increased from 2 to 6 h. These findings suggest clinical sampling of fluoroquinolone serum and CSF concentrations may be optimized by including both early and late samples. Due to their moderate lipophilic and protein bound nature in conjunction with relatively low molecular mass (Nau et al., 2010; Nau et al., 1994), fluoroquinolones enter CSF more completely than other antibiotics, with an estimated CSF concentration of approximately 20–80% of peak serum levels (Andes and Craig, 1999; Wilkinson et al., 2017). Our data support these published estimates, with median levofloxacin CSF concentrations ∼41% of total serum concentrations at 2 h and −63% at 6 h, and ofloxacin reaching −37% and −75% of total serum concentrations at 2 and 6 h post dose, respectively. Fluoroquinolone bacterial killing depends on concentration (both C_max_ and AUC), with a proposed C_max_/MIC ratio target of >8–10 in TB (Turnidge, 1999; Berning, 2001; Levison and Levison, 2009). Although we were unable to calculate CSF C_max_/MIC or AUC_0-24_/MIC for this population, fluoroquinolones in our study achieved concentrations within expected ranges, suggesting potential attainment of targets. Assuming susceptible MICs of 1 mg/L for ofloxacin and 0.5 mg/L for levofloxacin and moxifloxacin, levofloxacin may have the best potential to achieve PK/PD targets given our findings (Angeby et al., 2010). In addition, we found that levofloxacin CSF concentrations increased from baseline at both two and 6-h timepoints, supporting previous research that fluoroquinolones may adequately penetrate into the CNS regardless of inflammation (Andes and Craig, 1999; Wilkinson et al., 2017). The increase in CSF concentrations over time may be the result of drug accumulation and changes in the inflammatory state/blood brain barrier. These findings improve confidence that patients receiving fluoroquinolones for TBM can continue to have CSF drug exposure throughout therapy. Interestingly, while coadministration of moxifloxacin with rifampin has been associated with a reduction of moxifloxacin concentrations by approximately 26%–32% due to rifampin’s induction of cytochrome P450 enzymes, the present study did not find any statistically significant differences in moxifloxacin exposure (Nijland et al., 2007).

Although carbapenems have shown efficacy in bacterial meningitis and against Mtb in vitro and *in vivo* animal models (Chambers et al., 1995; Hugonnet et al., 2009; Veziris et al., 2011; Kaushik et al., 2015), this study found lower than expected CSF concentrations. While meropenem and imipenem had similar PK profiles to previous literature, median serum Cmax concentrations were below previously reported ranges after a 1-g dose (60–70 mg/L for imipenem and 50–60 mg/L for meropenem) (Zhanel et al., 2007). CSF penetration of imipenem and meropenem in previously published literature ranged from −16 to 41% of serum for imipenem and −11% for meropenem (Andes and Craig, 1999). In the present study, the majority of imipenem and meropenem CSF concentrations were either undetectable or BLQ. One potential contributor to our unexpected low CSF concentrations is that beta lactams may diffuse from the central nervous compartments to the blood during meningitis due to an increase in CSF pH, facilitating excretion of beta lactams (Nau et al., 2010; Thea and Barza, 1989). However, data suggests that brain tissue concentrations of meropenem may be sufficient, even in non-inflamed brain tissue (Hosmann et al., 2021). Techniques such as cerebral microdialysis or innovative molecular imaging methods are needed for a better understanding of the CNS penetration of carbapenems in the setting of infections such as TBM (Tucker et al., 2018)^,^ (Brunner et al., 2004).

While increased penetration of antimicrobials into the CNS is desired for treatment of TBM, this may be associated with increased risk of CNS-related adverse effects. Previous literature has described adverse CNS effects for fluoroquinolones and carbapenems, potentially due to interferences with gamma-aminobutyric acid receptors (Akahane et al., 1989; Wong et al., 1991; Hikida et al., 1993; Domagala, 1994; Sunagawa et al., 1995; Walton et al., 1997; Kushner et al., 2001; Owens and Ambrose, 2005; Mehlhorn and Brown, 2007; Kalita et al., 2016). Notably, while rate of seizures in patients receiving intravenous imipenem for bacterial meningitis has been reported as high as 33% (Wong et al., 1991), the safety margin is considered to be higher for meropenem (Norrby, 1996). It is promising that in this study, none of the participants experienced seizures.

There are limitations to this study, including a relatively small sample size of 22 patients. Also, due to the descriptive nature of the study, the impact of co-administered medications, renal function, and hepatic function were not controlled for in the analysis. However, expected impact is minimal. While we assumed the CSF samples with undetectable imipenem and meropenem concentrations were due to poor CSF penetration, we could not rule out other causes. The effect of carbapenem instability both before and after sample collection could not be assessed and may have contributed to the low CSF and serum concentrations, even though samples were rapidly stored at −80°C to help minimize degradation. For example, imipenem tested in conjunction with cilastatin only remained 90% stable for 2 h at 37°C or 3 h at 25°C, demonstrating its unstable nature prior to sample freezing (Viaene et al., 2002). With storage at temperatures below −20°C, the data on carbapenem stability is inconclusive (Gravallese et al., 1984; Myers and Blumer, 1984; Garcia-Capdevila et al., 1997). Previous literature suggests that meropenem undergoes degradation, even at −20°C, so it is possible unintentional degradation may have occurred during our sample collection, storage, or shipping processes (Gijsen et al., 2021). In addition to these limitations, CSF to serum ratios and PK/PD targets that can improve clinical outcomes in patients with TBM were not ascertained in this study. While these targets are not known, nor their effects on clinical outcomes, it is promising that 21/22 (95%) of participants in our study were able to complete therapy and only 1/22 (5%) had treatment failure. Also, the impact of co-administered medications, renal function, and hepatic function on drug concentrations was not assessed. Finally, the protein content and drug protein binding in CSF are not well established and the assumption of 0% protein binding in the CSF is based on limited data (Bonati et al., 1982; Nau et al., 2010).

In summary, we described fluoroquinolone and carbapenem penetration into the CSF in a cohort of patients treated for TBM. Our study is unique in that serial CSF concentrations were collected at 2 and 6 h over the first few months of TBM treatment, allowing for assessment of delayed penetration. We found that fluoroquinolones showed CSF penetration peaking after serum concentrations, potentially increasing over the duration of treatment. Carbapenems had lower than expected CSF concentrations, with the majority of samples undetectable or BLQ in the CSF while detectable in serum. These findings warrant further exploration with techniques such as cerebral microdialysis to see how carbapenem brain tissue concentrations compare to plasma and CSF concentrations.

## Data Availability

The raw data supporting the conclusions of this article will be made available by the authors, without undue reservation.

## References

[B1] Akahane K. Sekiguchi M. Une T. Osada Y. (1989). Structure-epileptogenicity relationship of quinolones with special reference to their interaction with gamma-aminobutyric acid receptor sites. Antimicrob. Agents Chemother. 33 (10), 1704–1708. 10.1128/AAC.33.10.1704 2556076 PMC172741

[B2] Alsultan A. Peloquin C. A. (2014). Therapeutic drug monitoring in the treatment of tuberculosis: An update. Drugs 74 (8), 839–854. 10.1007/s40265-014-0222-8 24846578

[B3] Andes D. R. Craig W. A. (1999). Pharmacokinetics and pharmacodynamics of antibiotics in meningitis. Infect. Dis. Clin. North Am. 13 (3), 595–618. 10.1016/s0891-5520(05)70096-9 10470557

[B4] Angeby K. A. Jureen P. Giske C. G. Chryssanthou E. SturEgard E. NordvallM. (2010). Wild-type MIC distributions of four fluoroquinolones active against *Mycobacterium tuberculosis* in relation to current critical concentrations and available pharmacokinetic and pharmacodynamic data. J. Antimicrob. Chemother. 65 (5), 946–952. 10.1093/jac/dkq091 20332195

[B5] Bartzatt R. (2011). Tuberculosis infections of the central nervous system. Cent. Nerv. Syst. Agents Med. Chem. 11 (4), 321–327. 10.2174/1871524911106040321 22384992

[B6] Berning S. E. (2001). The role of fluoroquinolones in tuberculosis today. Drugs 61 (1), 9–18. 10.2165/00003495-200161010-00002 11217874

[B7] Bolon M. K. (2009). The newer fluoroquinolones. Infect. Dis. Clin. North Am. 23 (4), 1027–1051. x. 10.1016/j.idc.2009.06.003 19909896

[B8] Bonati M. Kanto J. Tognoni G. (1982). Clinical pharmacokinetics of cerebrospinal fluid. Clin. Pharmacokinet. 7 (4), 312–335. 10.2165/00003088-198207040-00003 6749368

[B9] Brunner M. Langer O. Dobrozemsky G. Muller U. Zeitlinger M. Mitterhauser M. (2004). [18F]Ciprofloxacin, a new positron emission tomography tracer for noninvasive assessment of the tissue distribution and pharmacokinetics of ciprofloxacin in humans. Antimicrob. Agents Chemother. 48 (10), 3850–3857. 10.1128/AAC.48.10.3850-3857.2004 15388445 PMC521875

[B10] Chambers H. F. Moreau D. Yajko D. MiiCk C. Wagner C. HaCkbarth C. (1995). Can penicillins and other beta-lactam antibiotics be used to treat tuberculosis? Antimicrob. Agents Chemother. 39 (12), 2620–2624. 10.1128/AAC.39.12.2620 8592990 PMC163000

[B11] Christensen A. S. H. Roed C. Omland L. H. Andersen P. H. Obel N. Andersen Å. B. (2011). Long-term mortality in patients with tuberculous meningitis: A Danish nationwide cohort study. PLoS One 6 (11), e27900. 10.1371/journal.pone.0027900 22132165 PMC3222654

[B12] Craig W. A. (1997). The pharmacology of meropenem, a new carbapenem antibiotic. Clin. Infect. Dis. 24, S266–S275. 10.1093/clinids/24.supplement_2.s266 9126702

[B13] de Lange E. C. M. (2013). Utility of CSF in translational neuroscience. J. Pharmacokinet. Pharmacodyn. 40 (3), 315–326. 10.1007/s10928-013-9301-9 23400635 PMC3663203

[B14] Dheda K. Gumbo T. Maartens G. Dooley K. E. McNerney R. Murray M. (2017). The epidemiology, pathogenesis, transmission, diagnosis, and management of multidrug-resistant, extensively drug-resistant, and incurable tuberculosis. Lancet Respir. Med. 15S2213-2600 (17), 291–360. 10.1016/S2213-2600(17)30079-6 28344011

[B15] Domagala J. M. (1994). Structure-activity and structure-side-effect relationships for the quinolone antibacterials. J. Antimicrob. Chemother. 33 (4), 685–706. 10.1093/jac/33.4.685 8056688

[B16] Donald P. R. (2010). Cerebrospinal fluid concentrations of antituberculosis agents in adults and children. Tuberculosis 90 (5), 279–292. 10.1016/j.tube.2010.07.002 20709598

[B17] Garcia-Capdevila L. López-Calull C. Arroyo C. Moral M. A. Mangues M. A. Bonal J. (1997). Determination of imipenem in plasma by high-performance liquid chromatography for pharmacokinetic studies in patients. J. Chromatogr. B Biomed. Sci. Appl. 692 (1), 127–132. 10.1016/s0378-4347(96)00498-7 9187391

[B18] Ghimire S. Maharjan B. Jongedijk E. M. Kosterink J. G. W. Ghimire G. R. Touw D. J. (2019). Levofloxacin pharmacokinetics, pharmacodynamics and outcome in multidrug-resistant tuberculosis patients. Eur. Respir. J. 53 (4), 1802107. 10.1183/13993003.02107-2018 30655280

[B19] Gijsen M. Filtjens B. Annaert P. Armoudjian Y. Debaveye Y. Wauters J. (2021). Meropenem stability in human plasma at -20 °C: Detailed assessment of degradation. Antibiot. (Basel) 10 (4), 449. 10.3390/antibiotics10040449 PMC8072937 33923550

[B20] Gravallese D. A. Musson D. G. Pauliukonis L. T. Bayne W. F. (1984). Determination of imipenem (N-formimidoyl thienamycin) in human plasma and urine by high-performance liquid chromatography, comparison with microbiological methodology and stability. J. Chromatogr. 310 (1), 71–84. 10.1016/0378-4347(84)80069-9 6389581

[B21] Heemskerk A. D. Bang N. D. Mai N. T. H. Chau T. T. H. Phu N. H. Loc P. P. (2016). Intensified antituberculosis therapy in adults with tuberculous meningitis. N. Engl. J. Med. 374 (2), 124–134. 10.1056/NEJMoa1507062 26760084

[B22] Hikida M. Masukawa Y. Nishiki K. Inomata N. (1993). Low neurotoxicity of LJC 10, 627, a novel 1 beta-methyl carbapenem antibiotic: Inhibition of gamma-aminobutyric acidA, benzodiazepine, and glycine receptor binding in relation to lack of central nervous system toxicity in rats. Antimicrob. Agents Chemother. 37 (2), 199–202. 10.1128/AAC.37.2.199 8383938 PMC187638

[B23] Hosmann A. Ritscher L. Burgmann H. Al Jalali V. Wulkersdorfer B. Wolfl-Duchek M. (2021). Meropenem concentrations in brain tissue of neurointensive care patients exceed CSF levels. J. Antimicrob. Chemother. 76 (11), 2914–2922. 10.1093/jac/dkab286 34392352

[B24] Hugonnet J. E. Tremblay L. W. Boshoff H. I. Barry C. E. Blanchard J. S. (2009). Meropenem-clavulanate is effective against extensively drug-resistant *Mycobacterium tuberculosis* . Science 323 (5918), 1215–1218. 10.1126/science.1167498 19251630 PMC2679150

[B25] Huynh J. Donovan J. Phu N. H. Nghia H. D. T. Thuong N. T. T. Thwaites G. E. (2022). Tuberculous meningitis: Progress and remaining questions. Lancet. Neurol. 21 (5), 450–464. 10.1016/S1474-4422(21)00435-X 35429482

[B26] Jaganath D. Lamichhane G. Shah M. (2016). Carbapenems against *Mycobacterium tuberculosis*: A review of the evidence. Int. J. Tuberc. Lung Dis. 20 (11), 1436–1447. 10.5588/ijtld.16.0498 27776583

[B27] Janssen Pharmaceuticals, Inc (1996). Levaquin [package insert]. Titusville, NJ: Janssen Pharmaceuticals, Inc.

[B28] Kalita J. Bhoi S. K. Betai S. Misra U. K. (2016). Safety and efficacy of additional levofloxacin in tuberculous meningitis: A randomized controlled pilot study. Tuberc. (Edinb) 98, 1–6. 10.1016/j.tube.2016.01.004 27156611

[B29] Kaushik A. Makkar N. Pandey P. Parrish N. Singh U. Lamichhane G. (2015). Carbapenems and rifampin exhibit synergy against *Mycobacterium tuberculosis* and Mycobacterium abscessus. Antimicrob. Agents Chemother. 59 (10), 6561–6567. 10.1128/AAC.01158-15 26259792 PMC4576034

[B30] Kempker R. R. Smith A. G. C. Avaliani T. Gujabidze M. Bakuradze T. Sabanadze S. (2022). Cycloserine and linezolid for tuberculosis meningitis: Pharmacokinetic evidence of potential usefulness. Clin. Infect. Dis. 29, 682ciab992–689. 10.1093/cid/ciab992 PMC9464073 34849645

[B31] Kushner J. M. Peckman H. J. Snyder C. R. (2001). Seizures associated with fluoroquinolones. Ann. Pharmacother. 35 (10), 1194–1198. 10.1345/aph.10359 11675843

[B32] Lamp K. C. Bailey E. M. Rybak M. J. (1992). Ofloxacin clinical pharmacokinetics. Clin. Pharmacokinet. 22 (1), 32–46. 10.2165/00003088-199222010-00004 1559306

[B33] Levison M. E. Levison J. H. (2009). Pharmacokinetics and pharmacodynamics of antibacterial agents. Infect. Dis. Clin. North Am. 23 (4), 791–815. vii. 10.1016/j.idc.2009.06.008 19909885 PMC3675903

[B34] Litjens C. H. C. Aarnoutse R. E. Te Brake L. H. M. (2020). Preclinical models to optimize treatment of tuberculous meningitis - a systematic review. Tuberculosis 122, 101924. 10.1016/j.tube.2020.101924 32501258

[B35] Mehlhorn A. J. Brown D. A. (2007). Safety concerns with fluoroquinolones. Ann. Pharmacother. 41 (11), 1859–1866. 10.1345/aph.1K347 17911203

[B36] Myers C. M. Blumer J. L. (1984). Determination of imipenem and cilastatin in serum by high-pressure liquid chromatography. Antimicrob. Agents Chemother. 26 (1), 78–81. 10.1128/AAC.26.1.78 6591852 PMC179921

[B37] Nau R. Kinzig M. Dreyhaupt T. Kolenda H. Sörgel F. Prange H. W. (1994). Kinetics of ofloxacin and its metabolites in cerebrospinal fluid after a single intravenous infusion of 400 milligrams of ofloxacin. Antimicrob. Agents Chemother. 38 (8), 1849–1853. 10.1128/AAC.38.8.1849 7986019 PMC284648

[B38] Nau R. Sörgel F. Eiffert H. (2010). Penetration of drugs through the blood-cerebrospinal fluid/blood-brain barrier for treatment of central nervous system infections. Clin. Microbiol. Rev. 23 (4), 858–883. 10.1128/CMR.00007-10 20930076 PMC2952976

[B39] Nijland H. M. J. Ruslami R. Suroto A. J. Burger D. M. AlisjahBana B. van Crevel R. (2007). Rifampicin reduces plasma concentrations of moxifloxacin in patients with tuberculosis. Clin. Infect. Dis. 45 (8), 1001–1007. 10.1086/521894 17879915

[B40] Norrby S. R. (1996). Neurotoxicity of carbapenem antibacterials. Drug Saf. 15 (2), 87–90. 10.2165/00002018-199615020-00001 8884160

[B41] Ortho-McNeil-Janssen Pharmaceuticals, Inc (2008). Ofloxacin [package insert]. Raritan, NJ: Ortho-McNeil-Janssen Pharmaceuticals, Inc.

[B42] Owens R. C. Ambrose P. G. (2005). Antimicrobial safety: Focus on fluoroquinolones. Clin. Infect. Dis. 41, S144–S157. 10.1086/428055 15942881

[B43] Pickerill K. E. Paladino J. A. Schentag J. J. (2000). Comparison of the fluoroquinolones based on pharmacokinetic and pharmacodynamic parameters. Pharmacotherapy 20 (4), 417–428. 10.1592/phco.20.5.417.35062 10772373

[B44] Ramachandran G. Hemanth Kumar A. K. Srinivasan R. GeethArAni A. Sugirda P. Nandhakumar B. (2012). Effect of rifampicin & isoniazid on the steady state pharmacokinetics of moxifloxacin. Indian J. Med. Res. 136 (6), 979–984. 23391793 PMC3612327

[B45] Roos K. L. (2000). *Mycobacterium tuberculosis* meningitis and other etiologies of the aseptic meningitis syndrome. Semin. Neurol. 20 (3), 329–335. 10.1055/s-2000-9428 11051297

[B46] Salmon-Rousseau A. Martins C. Blot M. Mahy S. Chavanet P. Piroth L. (2020). Comparative review of imipenem/cilastatin versus meropenem. Med. Mal. Infect. 50 (4), 316–322. 10.1016/j.medmal.2020.01.001 32035719

[B47] Sharma S. K. Mohan A. Kohli M. (2021). Extrapulmonary tuberculosis. Expert Rev. Respir. Med. 15 (7), 931–948. 10.1080/17476348.2021.1927718 33966561

[B48] Smith A. G. C. Gujabidze M. Avaliani T. Blumberg H. M. Collins J. M. Sabanadze S. (2021). Clinical outcomes among patients with tuberculous meningitis receiving intensified treatment regimens. Int. J. Tuberc. Lung Dis. 25 (8), 632–639. 10.5588/ijtld.21.0159 34330348 PMC8443977

[B49] Sunagawa M. Matsumura H. Sumita Y. Nouda H. (1995). Structural features resulting in convulsive activity of carbapenem compounds: Effect of C-2 side chain. J. Antibiot. 48 (5), 408–416. 10.7164/antibiotics.48.408 7797443

[B50] Thea D. Barza M. (1989). Use of antibacterial agents in infections of the central nervous system. Infect. Dis. Clin. North Am. 3 (3), 553–570. 10.1016/s0891-5520(20)30289-0 2671139

[B51] Thwaites G. E. van Toorn R. Schoeman J. (2013). Tuberculous meningitis: More questions, still too few answers. Lancet. Neurol. 12 (10), 999–1010. 10.1016/S1474-4422(13)70168-6 23972913

[B52] Tucker E. W. Guglieri-Lopez B. Ordonez A. A. Ritchie B. Klunk M. H. Sharma R. (2018). Noninvasive 11C-rifampin positron emission tomography reveals drug biodistribution in tuberculous meningitis. Sci. Transl. Med. 10 (470), eaau0965. 10.1126/scitranslmed.aau0965 30518610 PMC6360528

[B53] Turnidge J. (1999). Pharmacokinetics and pharmacodynamics of fluoroquinolones. Drugs 58, 29–36. 10.2165/00003495-199958002-00006 10553702

[B54] van Rijn S. P. Zuur M. A. Anthony R. Wilffert B. van Altena R. Akkerman O. W. (2019). Evaluation of carbapenems for treatment of multi- and extensively drug-resistant *Mycobacterium tuberculosis* . Antimicrob. Agents Chemother. 63 (2), E01489-18. 10.1128/AAC.01489-18 30455232 PMC6355583

[B55] Veziris N. Truffot C. Mainardi J. L. Jarlier V. (2011). Activity of carbapenems combined with clavulanate against murine tuberculosis. Antimicrob. Agents Chemother. 55 (6), 2597–2600. 10.1128/AAC.01824-10 21402832 PMC3101399

[B56] Viaene E. Chanteux H. Servais H. Mingeot-Leclercq M. P. Tulkens P. M. (2002). Comparative stability studies of antipseudomonal beta-lactams for potential administration through portable elastomeric pumps (home therapy for cystic fibrosis patients) and motor-operated syringes (intensive care units). Antimicrob. Agents Chemother. 46 (8), 2327–2332. 10.1128/AAC.46.8.2327-2332.2002 12121900 PMC127357

[B57] Walton G. D. Hon J. K. Mulpur T. G. (1997). Ofloxacin-induced seizure. Ann. Pharmacother. 31 (12), 1475–1477. 10.1177/106002809703101206 9416384

[B58] Wilkinson R. J. Rohlwink U. Misra U. K. van Crevel R. Mai N. T. H. Dooley K. E. (2017). Tuberculous meningitis. Nat. Rev. Neurol. 13 (10), 581–598. 10.1038/nrneurol.2017.120 28884751

[B59] Wong V. K. Wright H. T. Ross L. A. Mason W. H. Inderlied C. B. Kim K. S. (1991). Imipenem/cilastatin treatment of bacterial meningitis in children. Pediatr. Infect. Dis. J. 10 (2), 122–125. 10.1097/00006454-199102000-00009 2062603

[B60] World Health Organization (2021). Global tuberculosis report 2021. Geneva: World Health Organization. (Accessed June 29, 2022).

[B61] Zhanel G. G. Wiebe R. Dilay L. Thomson K. Rubinstein E. Hoban D. J. (2007). Comparative review of the carbapenems. Drugs 67 (7), 1027–1052. 10.2165/00003495-200767070-00006 17488146

